# Constructing a Broad-Pore-Domain Structure of Adsorbents for Acteoside Adsorption

**DOI:** 10.3390/polym17010079

**Published:** 2024-12-31

**Authors:** Weibo Ru, Jiaxing Liu, Feng Xiong, Yu Sun, Yong Zhang, Yipei Li, Yin Lv, Xueqin Li

**Affiliations:** School of Chemistry and Chemical Engineering/State Key Laboratory Incubation Base for Green Processing of Chemical Engineering, Shihezi University, Shihezi 832003, China

**Keywords:** hyper-crosslinked polymer, mesopore, separation, bioactive component

## Abstract

Acteoside (ACT) is an important medicinal component, but its content is scarce. To obtain higher purity of ACT, the adsorption method was used to purify it. In this study, a broad-pore-domain hyper-crosslinked polymer (BHP-Kae) was prepared to adsorb ACT from *Cistanche tubulosa*, which is a medicinal plant. BHP-Kae-3 possessed a unique broad-pore-domain structure. This structure reduced the transfer resistance of ACT and facilitated the rapid diffusion of ACT into BHP-Kae-3, increasing the adsorption capacity. In addition, the surface and pore channels of BHP-Kae-3 contained abundant functional groups (-OH, C=O), which provided a large number of adsorption sites and facilitated ACT adsorption, thereby improving selectivity. The experimental results showed that BHP-Kae-3 exhibited a good adsorption capacity for ACT; the adsorption capacity was 105.12 mg/g, and the selectivity was 3.41. This study demonstrates the potential for efficient separation of natural products using broad-pore-domain adsorbents.

## 1. Introduction

*Cistanche tubulosa* is a perennial parasitic plant, which is widely distributed in Inner Mongolia, Ningxia, Gansu, Qinghai, and Xinjiang [1,2,3,4,5]. It is known for its distinctive flowers and unique method of obtaining nutrients by parasitizing other plants. The stems of *Cistanche tubulosa* are highly valued for their medicinal properties and were recorded in ancient Chinese medical literature such as *Shengnong*’s Classic of Materia Medica and *Compendium of Materia Medica* [6]. In recent times, the medicinal value of *Cistanche tubulosa* has been further explored through pharmacological studies [7]. *Acteoside* (ACT) (Figure S1) in *Cistanche tubulosa* has been found to possess a wide range of therapeutic effects [8]. These include neuroprotective properties [9,10], immune system modulation, anti-aging effects, prevention of osteoporosis, intestinal laxative properties, and liver protection [11,12,13,14,15,16]. There are various methods for extracting the active ingredients of ACT from *Cistanche tubulosa*. Conventional methods like chromatographic separation and extraction have been used [17,18], but they have shown limitations in terms of yield. For example, previous studies by Li et al. using high-speed counter-current chromatography obtained low yields of ACT [19]. Similarly, Nie et al. used deep eutectic solvent to extract ACT, and the yield was only 2.13 mg/g [20]. However, a more promising method for extracting bioactive components from natural products is adsorption [21]. This technique has been proven to be efficient, energy-saving, easy to operate, and environmentally friendly. Research using this method has the potential to improve ACT yield and purity.

The adsorption performance of an adsorbent primarily hinges on its physical and chemical structure [22,23,24]. Porous organic polymers are a novel type of porous material formed through covalent bonding of organic monomers [25,26]. Thanks to their excellent chemical and thermal stability, high specific surface area, and customizable pore structure, they have garnered significant attention in recent times [27]. In particular, hyper-crosslinked polymers possess the advantages of being highly stable [28,29], having a high specific surface area and low density [30], making them an incredibly desirable adsorbent [31,32]. Their stability ensures that they do not degrade in harsh environments or with prolonged use. Their high specific surface area allows for more adsorption sites, thereby increasing their adsorption capacity. Hyper-crosslinked polymers are easy to handle and transport due to their low density, which makes them lightweight [33]. From a synthesis standpoint, hyper-crosslinked polymers can be rapidly produced by a one-step Friedel–Crafts alkylation reaction, and the reaction uses inexpensive reagents and mild reaction conditions [34]. The production of hyper-crosslinked polymers not only reduces the production costs, but also minimizes the environmental effect associated with the use of hazardous reagents and energy-intensive processes [35]. Overall, the unique physical and chemical properties of hyper-crosslinked polymers and their ease of synthesis make them a highly attractive adsorbent for various applications, such as gas separation [36], water purification [37,38], and environmental remediation [39,40,41]. Researchers and industries are increasingly focusing on exploring the potential of hyper-crosslinked polymers and developing new techniques to further enhance their adsorption properties [28].

Kaempferol is a naturally occurring flavonol that contains high concentrations of phenolic hydroxyl groups [42,43], which contribute to its antioxidant properties [44]. Due to its polyphenol structure, it is an ideal monomer for the synthesis of hydrophilic hydroxyl hyper-crosslinked polymers (BHP-Kae) [45,46]. BHP-Kae, synthesized via a Friedel–Crafts alkylation reaction, possesses a broad-pore-domain structure. This unique structure is conducive to reducing the transfer resistance of ACT into BHP-Kae, facilitating rapid inward diffusion of ACT and improving the adsorption efficiency of BHP-Kae. Furthermore, the surface of BHP-Kae is characterized by the presence of hydroxyl groups and benzene rings. These functional groups have a significant effect on enhancing the interaction forces between BHP-Kae and ACT. This study also explored the effects of different adsorption conditions on the adsorption performance of BHP-Kae. The effects of pH [47], temperature [48], and adsorption time on the adsorption process were investigated [49]. The findings of this study provide valuable insights and guidance for future research in this field.

## 2. Experimental Section

**Figure 1:**
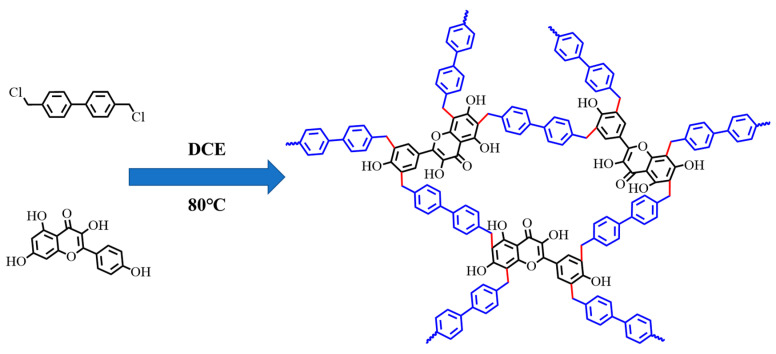
Illustration of BHP-Kae synthesis.

BHP-Kae was synthesized using the Friedel–Crafts method. First, 0.18 g of kaempferol and 0.48 g of 4,4′-bis(chloromethyl)-1,1′-biphenyl were mixed with 40 mL of 1,2-dichloroethane and sonicated to dissolve completely. Next, 0.30 g of iron (III) chloride was added to the mixture and then heated at 80 °C for a duration of 20 h while being agitated to complete polymerization. After completion of the reaction, it was cooled to room temperature and the solid product was collected through centrifugation and washed three times with ultra-pure water. Finally, methanol was employed as the extraction solvent for Soxhlet extraction. The resulting product was dried under vacuum at 50 °C, resulting in the formation of a dark green solid powder, which was designated as BHP-Kae-3 (Figure 1).

To examine the effect of reaction time on the adsorption capability of the adsorbent, BHP-Kae-1, BHP-Kae-2, and BHP-Kae-4 were also synthesized following the same procedure as BHP-Kae-3, but with different reaction times of 4 h, 12 h, and 28 h, respectively.

## 3. Results and Discussion

### 3.1. Characterization of BHP-Kae

**Figure 2:**
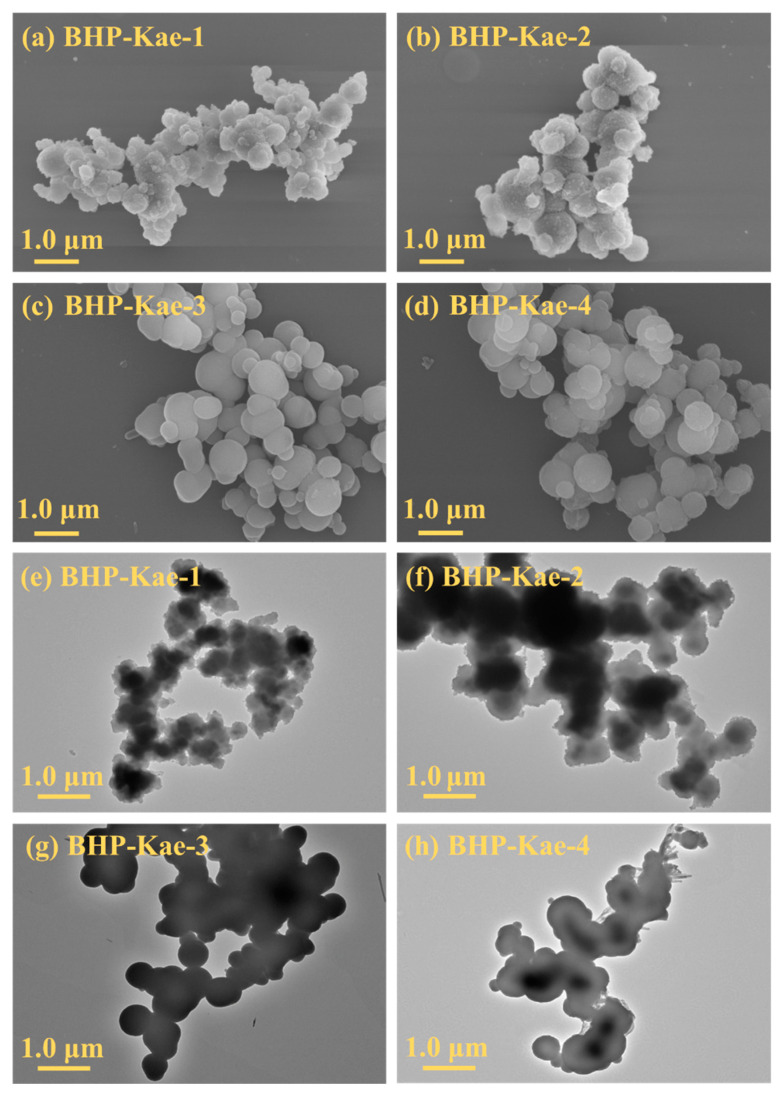
SEM (**a**–**d**) and TEM (**e**–**h**) images of BHP-Kae.

Figure 2 displays scanning electron microscope (SEM) and transmission electron microscope (TEM) images of BHP-Kae at different reaction times. The images revealed that BHP-Kae possessed a rough microsphere structure, with spheres ranging in diameter from 300 nm to 1 μm. Figure 2a,b,e,f show rough microsphere structures, which are caused by short reaction times and incomplete crosslinking. Moreover, the surfaces of these microspheres became smoother as the reaction time increased, indicating that the microstructure of BHP-Kae could be adjusted by manipulating the reaction time. Figure 2c,g provide evidence that when the reaction time reached 20 h, BHP-Kae-3 exhibited a grape-like structure. Figure 2d,h show that BHP-Kae-4 displayed irregular clusters, which were attributed to a prolonged reaction time and higher degree of crosslinking. It could be concluded that controlling the reaction time can adjust the structure of BHP-Kae.

**Figure 3:**
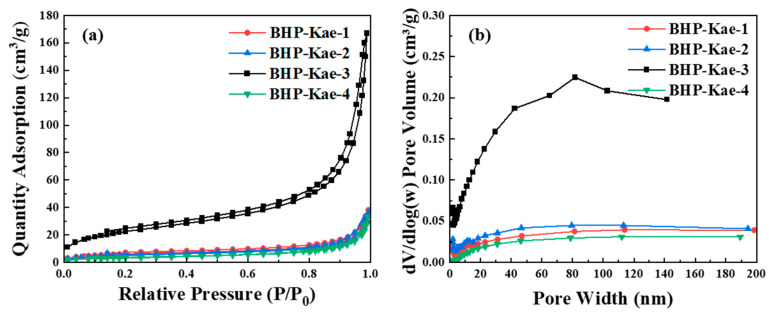
N_2_ adsorption and desorption isotherms (**a**), and pore size distributions (**b**) of BHP-Kae.

The adsorption–desorption curve and pore size distribution of the adsorbent BHP-Kae were observed, as shown in Figure 3. In Table S1, the specific surface area and pore structure parameters of BHP-Kae are given. From the observation of Figure 3a, the adsorption–desorption curve for BHP-Kae was classified as Type IV, and it showed distinct H3 hysteresis loops. From these characteristics, it could be inferred that BHP-Kae was a mesoporous material. The specific surface area and pore size of BHP-Kae varied depending on the duration of the reaction. Figure 3b shows the broad-pore-domain structure of BHP-Kae-3, and this structure can effectively reduce the transfer resistance when ACT enters BHP-Kae. The molecules inside BHP-Kae could be rapidly and inwardly diffused as well, which would help improve adsorption efficiency.

Based on the parameters shown in Table S1, it was observed that the specific surface area and pore volume of BHP-Kae initially increased and then decreased as the reaction time increased. Among the samples, BHP-Kae-3 exhibited the highest specific surface area of 82.36 m^2^/g and the largest pore size of 17.07 nm. June et al. [50] reported that HCP-Fe was 64 m^2^/g, and this difference may be related to the sample treatment method in the experiment. The high specific surface area and large pore size of BHP-Kae-3 could be attributed to a couple of reasons. On the one hand, when the crosslinking time was either too long or too short, it hindered the formation of microspheres, leading to a decrease in the specific surface area. On the other hand, excessive crosslinking led to pore clogging, which reduced the specific surface area and pore volume. Theoretically, the high specific surface area and large pore volume provided a significant number of adsorption sites. This facilitated transfer and improved the adsorption performance of BHP-Kae from *Cistanche tubulosa*.

**Figure 4:**
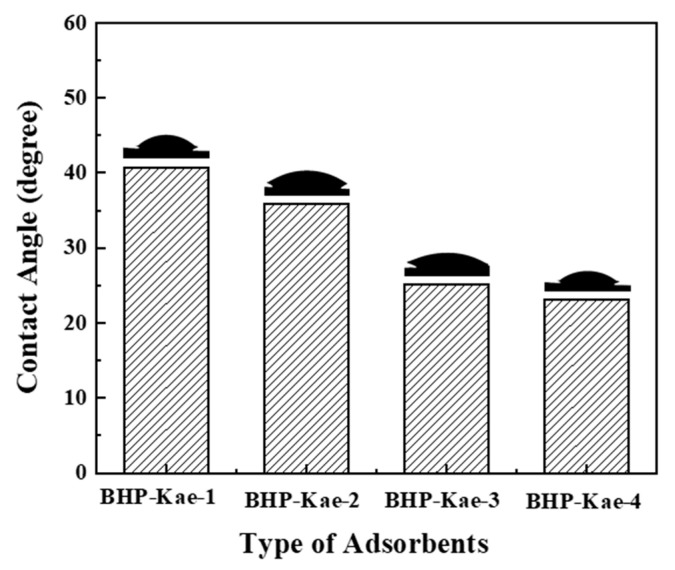
Water contact angle of BHP-Kae.

To investigate the effect of different reaction times on the hydrophilicity of BHP-Kae, water contact angle tests were carried out on the adsorbent. The test results are displayed in Figure 4. It was observed that the water contact angle of BHP-Kae gradually decreased from 40.1° to 23.2° as the reaction time increased from 4 h to 28 h. Significantly, when the reaction time reached 28 h, the water contact angle of BHP-Kae-4 hit its lowest point. This decline in the water contact angle could be attributed to the presence of abundant phenolic hydroxyl groups on the surface of BHP-Kae. These phenolic hydroxyl groups greatly contributed to the hydrophilicity of BHP-Kae, making it exhibit a strong affinity towards water. These findings highlight the importance of reaction time in determining the hydrophilicity of the BHP-Kae adsorbent. Consequently, the adsorbent became more and more hydrophilic, as evidenced by the progressive decrease in the water contact angle.

**Figure 5:**
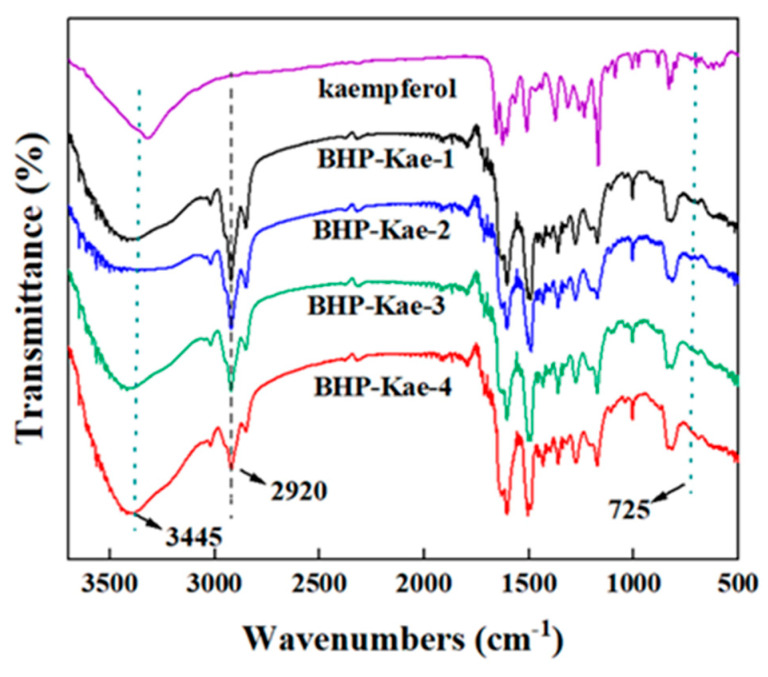
FTIR spectra of BHP-Kae.

In Figure 5, the Fourier transform infrared spectroscopy (FTIR) of BHP-Kae is shown. The prepared BHP-Kae exhibited a distinctive peak at 3445 cm^−1^, indicating the presence of the -OH group on kaempferol. This peak confirmed that the -OH group was not consumed during the crosslinking process. Additionally, a new peak appeared at 2920 cm^−1^, which was attributed to the stretching vibration of the -CH- bonds on the benzene ring. This peak indicated that the benzene ring was successfully incorporated into the structure of BHP-Kae. Moreover, the disappearance of the characteristic peak at 725 cm^−1^ indicated that the C-Cl bond in 4,4′-bis(chloromethyl)-1,1′-biphenyl was substituted by the benzene ring, resulting in the embedding of the two aromatic rings into the polymer backbone. These findings confirm the successful occurrence of the crosslinking reaction and the successful preparation of BHP-Kae.

**Figure 6:**
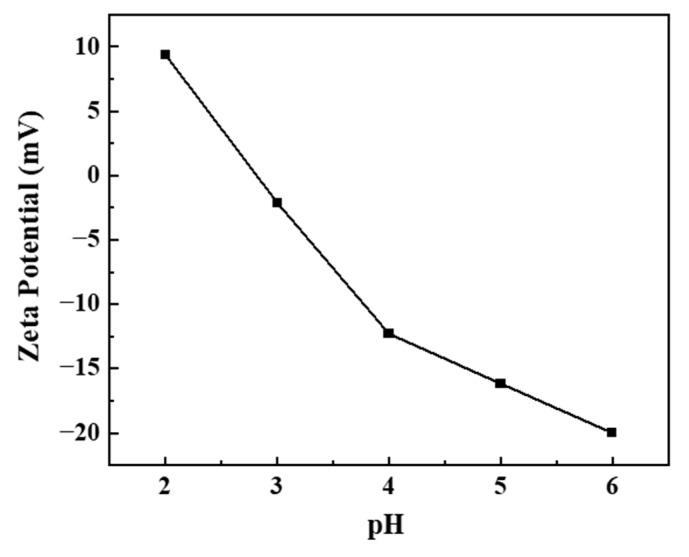
Zeta potential of BHP-Kae-3 at different pH.

Figure 6 presented the zeta potential, which measured the surface charge of BHP-Kae-3 under different pH conditions. The graph revealed that the zeta potential gradually decreased as the pH increased from 2 to 6. This was the same as the experimental results of Li et al. [51] and Wang et al. [52]; in a neutral environment, the zeta potential was negative. Moreover, the zero charge point (pH_ZPC_) of BHP-Kae-3 was precisely calculated to be 2.82. This meant that at a pH of 2.82, the surface charge of BHP-Kae-3 was neutral. In general, a zero charge adsorbent was advantageous for the formation of hydrogen bonds between the adsorbent and ACT. This was because a neutral adsorbent could establish a strong interaction through hydrogen bonding. Conversely, negatively charged adsorbents tended to exhibit an electrostatic attraction to positively charged target molecules, and vice versa. Notably, in the context of this study, ACT was an acidic molecule, but the zeta potential of the ACT solution at this time was −1.2 mV. During the adsorption process of BHP-Kae-3, the electrostatic interaction was found to be comparatively weak. Instead, the primary force responsible for adsorption was the formation of hydrogen bonds.

### 3.2. Adsorption Evaluation

#### 3.2.1. Effect of Adsorbent Reaction Time

**Figure 7:**
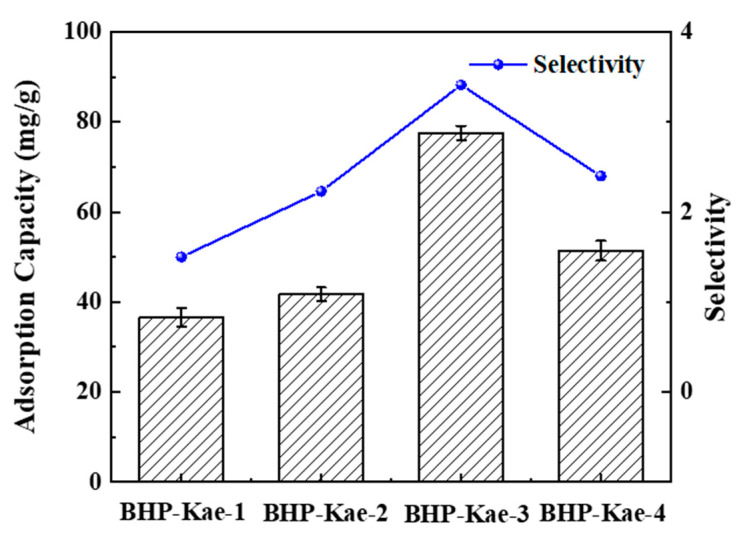
Adsorption performance of the BHP-Kae obtained at different reaction times.

The results depicted in Figure 7 provide insights into the adsorption capacity and selectivity of BHP-Kae for ACT at different reaction times (BHP-Kae-1, BHP-Kae-2, BHP-Kae-3, and BHP-Kae-4 correspond to 4 h, 12 h, 20h, and 28 h respectively). (High performance liquid chromatography (HPLC) was used for analysis, and HPLC was used for gradient elution. The elution conditions were set as showed in Table S2. The fitting standard curves of ECH and ACT obtained by HPLC are shown in Table S3. The HPLC diagrams of echinacoside (ECH) and ACT were showed in Figure S2.) They clearly demonstrate that the adsorption capacity and selectivity of BHP-Kae for ACT displayed an initial increment followed by a subsequent decrement as the reaction time increased. It is noteworthy that BHP-Kae-3 exhibited the most favorable adsorption performance for ACT, the adsorption capacity of 77.52 mg/g and a selectivity of 3.41 among the adsorbents examined. This superior performance could be attributed to two main reasons. On the one hand, BHP-Kae-3 possessed a broad-pore-domain structure. This unique pore structure facilitated the fast inward diffusion of molecules, thereby enhancing the monolithic transferred capacity of the adsorbent. On the other hand, BHP-Kae-3 contained hydroxyl and benzene rings, which interacted with ACT through multiple forces of interaction. These multiple interactions further enhanced the selective adsorption of ACT. The hydroxyl groups and benzene rings in BHP-Kae formed various types of interactions (hydrogen bonding, Van der Waals force and π–π interactions) with ACT, resulting in an improvement in the adsorption performance of BHP-Kae-3. Therefore, the following studies were all aimed at the adsorption evaluation of BHP-Kae-3.

#### 3.2.2. Effect of ACT Concentration

**Figure 8:**
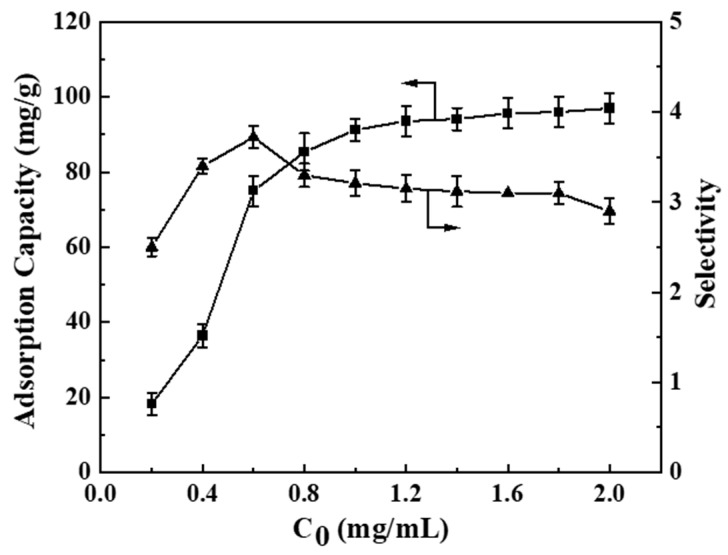
Effect of initial concentrations of ACT on BHP-Kae-3 adsorption performance.

Figure 8 depicts the influence of various initial concentrations on the adsorption performance of BHP-Kae-3. The observed trend indicated that the adsorption capacity of BHP-Kae-3 for ACT consistently rose as the concentration of ACT increased until it reached a point of adsorption equilibrium. As the concentration of ACT in the adsorption solution gradually increased, the number of ACT present also increased. It should be noted that the adsorption sites available on the surface of BHP-Kae-3 were limited. Therefore, the number of ACT occupying the adsorption sites on BHP-Kae-3 also increased as the concentration of ACT continued to rise. This led to a gradual accumulation of ACT on the BHP-Kae-3 until all the adsorption sites became fully occupied.

#### 3.2.3. Effect of pH

**Figure 9:**
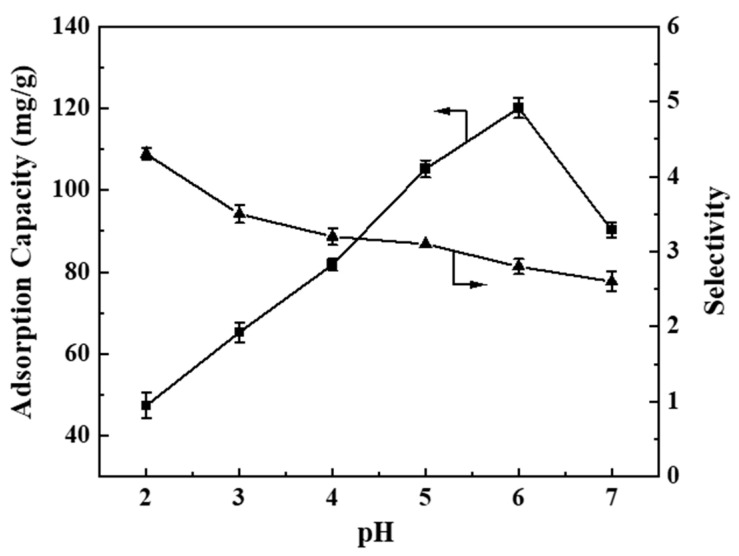
Effect of pH on BHP-Kae-3 adsorption performance.

Figure 9 illustrates the adsorption performance of BHP-Kae-3 for ACT under various pH. The pH of the solution directly influenced the stability of ACT and the formation of hydrogen bonds between the adsorbent and ACT. ACT is an acidic molecule that requires specific pH conditions to maintain its stability in solution. Therefore, it is crucial to control the pH of the solution within the range of 2 to 7. Further analysis of Figure 9 shows that altering the pH had a significant effect on the adsorption process of the adsorbent. The adsorption capacity of BHP-Kae-3 for ACT increased initially and then decreased with the increase in pH. Considering both the adsorption capacity and selectivity of BHP-Kae-3 for ACT, it was found that the optimal pH value of the adsorption solution was 5.

The reason behind this phenomenon is that the adsorbed solution had a strong polarity at pH = 2. This strong polarity inhibited the formation of hydrogen bonds between BHP-Kae-3 and ACT. In the absence of hydrogen bonding, BHP-Kae-3 had a lower adsorption capacity for ACT. The polarity of the adsorbed solution was weakened as the pH value increased, allowing BHP-Kae-3 and ACT to form stronger interactions. These hydrogen bonds facilitated the adsorption process, which led to a higher adsorption capacity of BHP-Kae-3 for ACT. This suggests that the adsorbent possessed enhanced adsorption capacity towards ACT and effectively captured more ACT at pH = 5.

#### 3.2.4. Influence of Adsorption Temperature

**Figure 10:**
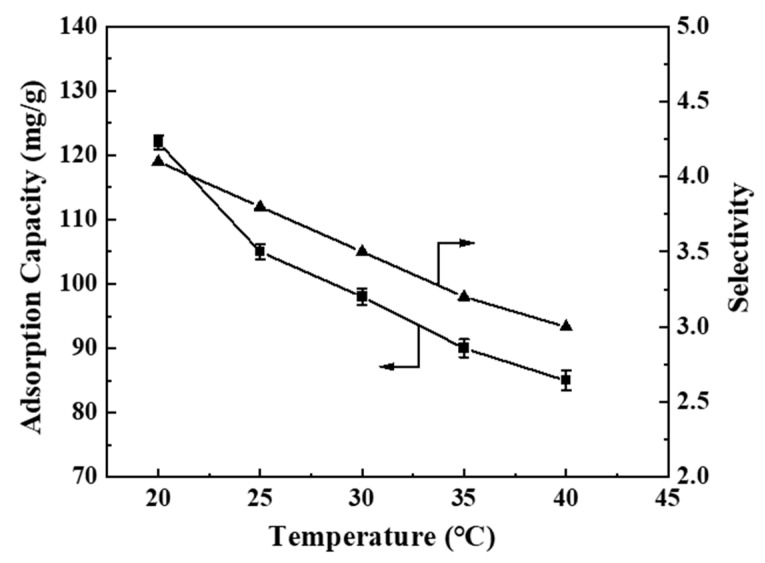
Effect of adsorption temperature on BHP-Kae-3 adsorption performance.

Figure 10 illustrates the adsorption performance of BHP-Kae-3 for ACT at various temperatures. The figure clearly shows that as the temperature increased from 20 °C to 40 °C, the adsorption capacity and selectivity gradually decreased. This decline could be attributed to the fact that the adsorption process of BHP-Kae-3 for ACT was an exothermic reaction, meaning that lower temperatures were more favorable for the adsorption of ACT by BHP-Kae-3. Considering the desired adsorption capacity and selectivity, it could be concluded that the optimal temperature for adsorption was 20 °C.

### 3.3. Study on Adsorption Kinetics of BHP-Kae-3

**Figure 11:**
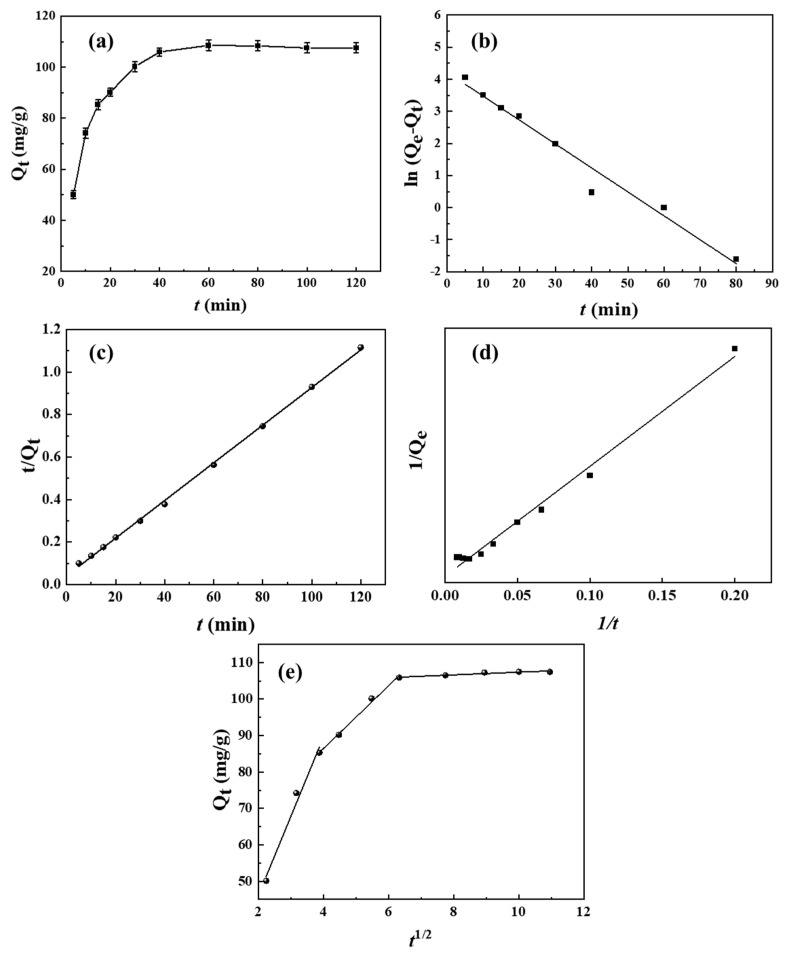
Adsorption kinetics of ACT on BHP-Kae-3: adsorption time (**a**), pseudo-first-order model (**b**), pseudo-second-order model (**c**), Ritchie-second-order model (**d**), and intra-particle diffusion model (**e**). (Q_t_ (mg/g) and Q_e_ (mg/g) represent the adsorption capacity at t (min) and the adsorption capacity at equilibrium, respectively).

A series of experiments were conducted to further explore the adsorption kinetics of BHP-Kae-3 for ACT. Upon analyzing Figure 11a, it was observed that the adsorption capacity of BHP-Kae-3 for ACT gradually increased with the passage of time until it reached the equilibrium state. Based on the experimental findings, it was found that the adsorption of BHP-Kae-3 for ACT reached equilibrium after approximately 50 min. It could be concluded that the adsorption of BHP-Kae-3 for ACT was a rapid adsorption process.

The results shown in Figure 11b–d and Table S4 clearly illustrate that the theoretical adsorption capacity predicted by the pseudo-second-order kinetic model closely matched the experimental value. Additionally, the correlation coefficient of the pseudo-second-order kinetic model was higher than that of the pseudo-first-order model and Ritchie-second-order [53,54] kinetic model. Based on these findings, it could be concluded that the pseudo-second-order kinetic model was more suitable and reliable for characterizing the adsorption phenomenon of BHP-Kae-3 in ACT.

Figure 11d and Table S5 illustrate the adsorption of BHP-Kae-3 for ACT, depicted by fitting the curve with the intra-particle diffusion model. The curve obtained from the experimental data did not intersect the origin, indicating that there was no singular rate-limiting step in the adsorption process. The adsorption of ACT by BHP-Kae-3 consisted of three distinct stages. The first stage was known as surface diffusion control stage, in which ACT diffused from the adsorbed solution to the BHP-Kae-3 surface to complete the surface adsorption process. The second stage was governed by intra-particle diffusion, in which ACT penetrated from the surface to the interior of BHP-Kae-3 through its broad-pore-domain structure. The third stage represented the equilibrium stage of adsorption and desorption. In this stage, the diffusion rate gradually decreased until it reached zero, signifying the attainment of equilibrium between the adsorption and desorption processes. Based on the adsorption kinetics observed for BHP-Kae-3, it could be concluded that the adsorption of ACT by BHP-Kae-3 was a rapid process.

### 3.4. Thermodynamic Study on Adsorption of BHP-Kae-3

**Figure 12:**
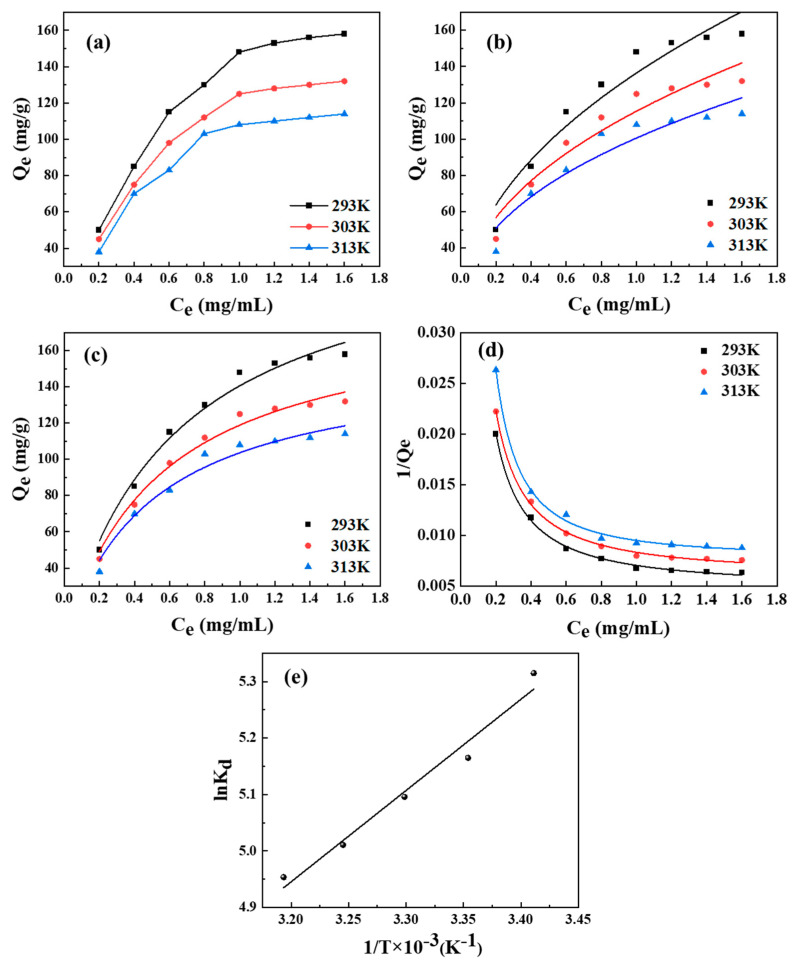
Adsorption isotherms of ACT on BHP-Kae-3: adsorption isotherms (**a**), Freundlich isotherm model (**b**), Langmuir isotherm model (**c**), Liu isotherm model (**d**), and thermodynamics of adsorption (**e**). (Q_e_ (mg/g) represents the equilibrium adsorption capacity of the adsorbent for ACT. C_e_ (mg/mL) represents the equilibrium concentration. K_d_ represents the partition coefficient).

Figure 12 investigates the adsorption phenomenon of BHP-Kae-3 for ACT through thermodynamic studies. The adsorption capacity of BHP-Kae-3 also gradually increased until it reached a state of equilibrium as the initial concentration of ACT increased, as shown in Figure 12a. Furthermore, it was evident from different adsorption temperatures that the adsorption capacity of BHP-Kae-3 for ACT decreased as the adsorption temperature increased. This suggested that the adsorption process was exothermic during the adsorption of ACT into BHP-Kae-3. The results revealed that the higher initial concentrations and the lower temperature led to increased adsorption capacity.

To study the adsorption process of BHP-Kae-3 for ACT in more detail, the Freundlich, Langmuir, and Liu [55] isotherm models were employed to fit the adsorption isotherm data (Figure 12b–d, Table S6). Analyzing the fitted data revealed that the Liu isotherm model yielded a higher correlation coefficient value compared to the Freundlich isotherm and Langmuir isotherm models at each adsorption temperature. Furthermore, the adsorption capacity calculated using the Liu isotherm model exhibited closer agreement with the experimental value. Consequently, the Liu isotherm model could describe the adsorption process of BHP-Kae-3 on ACT.

In order to further study the adsorption thermodynamics of the adsorption process, the change in enthalpy (ΔH), the change in Gibbs free energy (ΔG), and the change in entropy (ΔS) were calculated to explore the adsorption thermodynamics. The magnitude of ΔH was less than 40 kJ/mol, indicating that the adsorption process was predominantly governed by weak intermolecular forces and was a physical adsorption process. The calculated value of ΔG was negative and exhibited a decreasing trend with increasing temperature. This indicated that the surface adsorption process was spontaneous. Furthermore, the positive value of ΔS indicated that the affinity of BHP-Kae-3 for ACT increased during adsorption (Table S7). This further supported the spontaneous and exothermic physical adsorption process for the adsorption of ACT by BHP-Kae-3.

### 3.5. Adsorption Mechanism

#### 3.5.1. XPS Analysis

**Figure 13:**
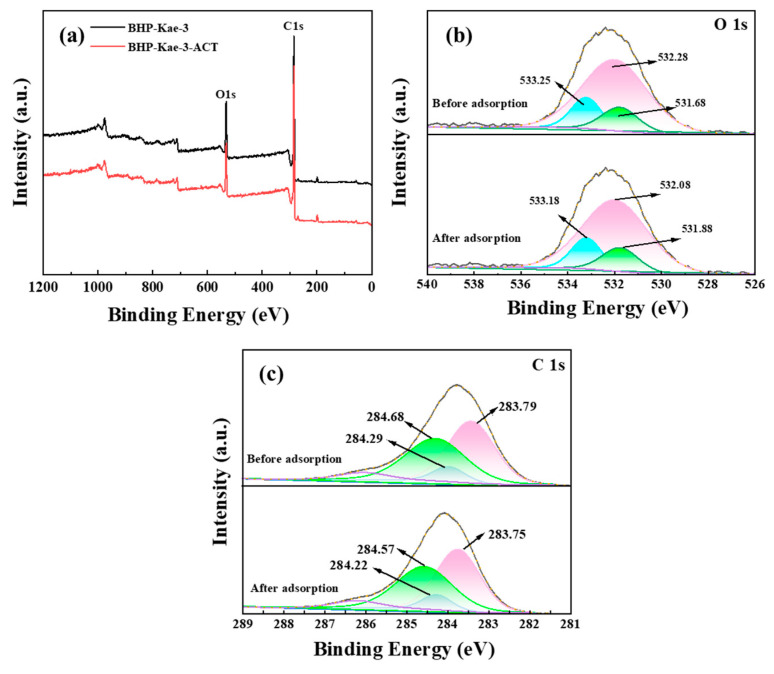
XPS spectra of BHP-Kae-3 before and after adsorption ACT: wide scans (**a**), O 1s (**b**), C 1s (**c**).

Figure 13 presents the XPS spectra of BHP-Kae-3 before and after the ACT adsorption process. In Figure 13a, it is evident that the peak value of the oxygen element in the adsorbent BHP-Kae-3-ACT shifted in energy after ACT adsorption, indicating the formation of hydrogen bonds between BHP-Kae-3 and ACT.

In Figure 13b, the XPS spectra of O 1s of BHP-Kae-3 and BHP-Kae-3-ACT are presented. Before the adsorption process, there was a prominent peak observed at 533.25 eV, indicating the presence of hydroxyl (-OH) groups on the BHP-Kae-3 surface. After the adsorption of ACT, the peak shifted slightly to 533.18 eV. This shift suggests that a hydrogen bonding interaction had occurred between the oxygen atom on BHP-Kae-3 and ACT.

Figure 13c presented XPS spectra of C 1s for two samples, BHP-Kae-3 and BHP-Kae-3-ACT. The spectrum of BHP-Kae-3 displayed three distinct peaks at 284.29, 284.68, and 283.79 eV, which corresponded to different carbon–oxygen (C-O), carbon–carbon (C-C), and carbon–carbon double bond (C=C) functionalities present in the benzene ring. The C-O peak shifted to 284.22 eV, the C-C peak shifted to 284.57 eV, and the C=C peak shifted to 283.75 eV. These shifts could be attributed to the formation of hydrogen bonds and π–π interactions between the adsorbent and ACT during the adsorption process. This information is visually represented in Figure S3, illustrating each force involved in the adsorption phenomenon.

#### 3.5.2. Molecular Simulation

**Figure 14:**
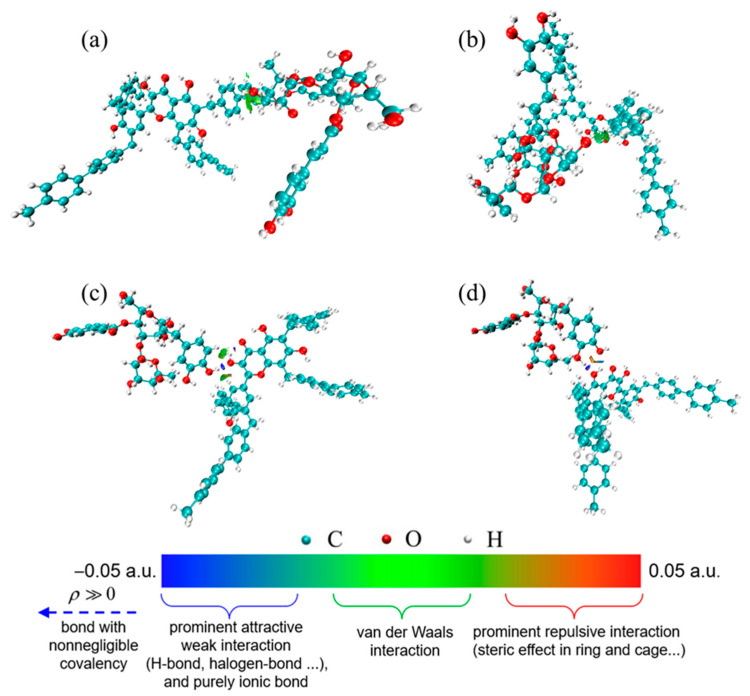
IGM analysis of binding configurations between fragments on BHP-Kae-3 and ACT: (**a**–**d**) four different locations in the BHP-Kae-3 building unit are combined with ACT.

In order to fully understand the interaction between BHP-Kae-3 and ACT, DFT calculations (Gaussian 09W) were used to explain the mechanism of their interaction. The optimal binding configuration and interaction patterns of BHP-Kae-3 and ACT were determined by DFT calculations, as shown in Figure 14. A unit on BHP-Kae-3 was chosen for the calculation because it could provide binding sites during the adsorption process and interact with ACT. In addition, the complexes were analyzed by independent gradient modeling (IGM) using Multiwfn software (Multiwfn_3.8_dev_bin_Win64) [56,57,58]. It was observed that hydrogen bonding and van der Waals interactions occurred simultaneously during the adsorption process. This was consistent with the results of the XPS analysis.

### 3.6. Study on Regeneration Ability of BHP-Kae-3

**Figure 15:**
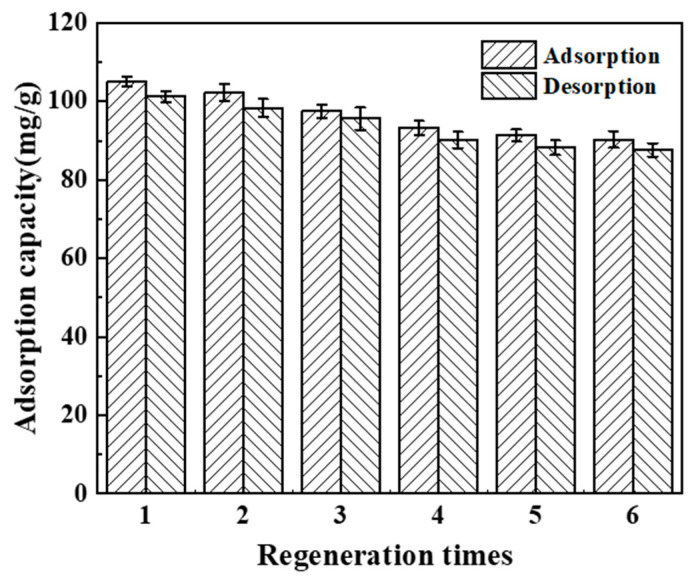
Adsorption–desorption cycle experiments of ACT on BHP-Kae-3.

The regeneration ability of BHP-Kae-3, a material used for practical applications, was found to be highly important. In order to evaluate its performance, the change in the adsorption capacity of BHP-Kae-3 for ACT was analyzed over the course of eight adsorption–desorption cycles, as depicted in Figure 15. According to the data presented in the figure, it can be observed that after BHP-Kae-3 was subjected to eight consecutive experiments, the adsorption capacity for ACT decreased from an initial value of 105.12 mg/g to 87.66 mg/g. Nonetheless, even after these cycles, the adsorption capacity of BHP-Kae-3 remained above 83% of its original value. Table S8 compared the adsorption capacity of BHP-Kae-3 and other previously reported adsorbents for ACT. Compared with other adsorbents, BHP-Kae-3 showed the maximum adsorption capacity and selectivity for ACT. From these results, one can confidently infer that BHP-Kae-3 possesses excellent regeneration ability and is therefore a suitable material for the adsorption of ACT.

## 4. Conclusions

In this study, a series of BHP-Kae adsorbents were successfully synthesized using the Friedel–Crafts reaction method. This method allowed adsorbents to effectively adsorb ACT. The pore structure of the BHP-Kae adsorbents was adjusted by controlling the reaction time. Compared with BHP-Kae-1, BHP-Kae-2, and BHP-Kae-4, the pore size of BHP-Kae-3 increased by 8.2%, 2.3%, and 1.7%, the pore volume increased by 1914.3%, 1310.0%, and 74.1%, and the specific surface area increased by 342.3%, 290.0%, and 150.9%, respectively. Specifically, when the reaction time was 20 h, the resulting BHP-Kae-3 exhibited a broad-pore-domain structure. Adsorption reached equilibrium quickly because the broad-pore-domain structure facilitated fast intra-molecular diffusion by reducing transfer resistance. Moreover, the surface of BHP-Kae-3 was rich in hydroxyl groups and benzene rings. These chemical groups possessed the ability to interact with ACT through multiple forces, including hydrogen bonding and π–π interactions. In addition, BHP-Kae-3 demonstrated excellent regeneration performance. The experimental results showed that, compared with BHP-Kae-1, BHP-Kae-2, and BHP-Kae-4, the adsorption capacity of BHP-Kae-3 increased by 111.7%, 85.8%, and 50.9%, and the selectivity was increased by 127.3%, 52.9%, and 42.1%, respectively. Broad-pore-domain adsorbents, as effective adsorbents, open up new possibilities for the extraction and purification of other bioactive compounds.

## Data Availability

Data are contained within the article and supplementary materials. Further inquiries can be directed to the corresponding author.

## Supplementary Materials

The following supporting information can be downloaded at: https://www.mdpi.com/article/10.3390/polym17010079/s1, Figure S1: the structural formula of ACT; Figure S2: HPLC chromatogram of ACT and ECH; Figure S3, the main interaction between BHP-Kae-3 and ACT; Table S1, specific surface area parameters and pore structure of BHP-Kae; Table S2, the gradient elution condition of HPLC; Table S3, the calibration curves of ACT and ECH; Table S4, kinetics parameters of ACT adsorption on BHP-Kae-3; Table S5, intra-molecule diffusion coefficient of ACT on BHP-Kae-3; Table S6, adsorption isotherm parameters for BHP-Kae-3; Table S7, thermodynamic parameters of ACT adsorption; Table S8, comparing the adsorption capacities of various ACT adsorbents [19,20,59,60,61].
