# Supplementary material for: Constructing a Broad-Pore-Domain Structure of Adsorbents for Acteoside Adsorption
Source: Polymers (Basel). 2024 Dec 31;17(1):79. doi: 10.3390/polym17010079 (PMC11722942; doi:10.3390/polym17010079)
Supplement: Supplementary file 1 [file polymers-17-00079-s001.zip › polymers-3378081-supplementary.pdf]

Supplementary materials

Constructing broad-pore-domain structure of adsorbents for acteoside adsorption

Weibo Ru, Jiaxing Liu, Feng Xiong, Yu Sun, Yong Zhang, Yipei Li, Yin Lv, Xueqin Li**

School of Chemistry and Chemical Engineering/State Key Laboratory Incubation Base for Green Processing of Chemical Engineering, Shihezi University, Shihezi 832003, China

Corresponding author

*X. Li. E-mail address: lixueqin861003@163.com.

*Y. Lv. E-mail address: ag_125@163.com

S1. Materials

Acteoside (ACT) and *echinacoside* (ECH) were obtained from Chengdu Desite Biological Technologies Co. Ltd. Acetic acid glacial, acetonitrile, methanol, kaempferol, 4,4'-bis(chloromethyl)-1,1'-biphenyl, 1, 2-dichloroethane (DCE), and Iron (III) chloride were purchased from Adamas (Shanghai, China). These reagents were of analytical purity and did not require further purification prior to use. Ultrapure water was used throughout the experiment process.

S2. Instruments

Scanning electron microscope (SEM, JEOL JSM-6460LV) and transmission electron microscope (TEM, FEI Tecnai G^2 F20) were used to observe the microstructure of BHP-Kae-3. The chemical composition of BHP-Kae-3 was detected in the range of 4000 to 500 cm^{-1} by Fourier transform infrared spectroscopy (FTIR, Bruker Optics, Ltd., Germany). The specific surface area and pore structure parameters of BHP-Kae-3 were estimated by the adsorption-desorption isotherms of N_2, and it used Brunauer-Emmet-Teller (BET, Micromeritics ASAP 2460) and Barret–Joyner–Halenda methods. The surface charge of BHP-Kae-3 was determined at the different pH values of adsorption solution using the zeta potential analyzer (NanoPLUS-3, Micromeritics Instrument Corp, USA). In addition, X-ray photoelectron spectroscopy (XPS, Thermo, USA) was used to analyze the adsorption mechanism of this adsorption process between BHP-Kae-3 and ACT. Hydrophilicity of BHP-Kae-3 was tested by water contact angle test (KRUSS DSA100S, Germany). The ground oscillator (HZQ-311C, China) oscillates the adsorption of BHP-Kae-3

for ACT.

S3. High performance liquid chromatography (HPLC) analysis

S3.1. Preparation of standard solution

Weigh ECH standard and ACT standard 20 mg each and put into 5 mL volumetric bottle. The mixture of methanol and water was dissolved and dispersed by ultrasound, and then stored in refrigerator for later use.

S3.2. Preparation of sample solution

The crude extract of *Cistanche tubulosa* was added to ultra-pure water, dispersed and dissolved by ultrasound, and stored in the refrigerator for future use.

S3.3. High performance liquid chromatography (HPLC) detection

The adsorption samples were detected by high performance liquid chromatography (HPLC, 2695, Waters Co., USA) was used to analyze the concentrations of ECH and ACT in the samples before and after adsorption with a C18 column using an ultraviolet detector. The detection conditions were that the wavelength of the UV detector was 330 nm and the temperature was 303 K. The gradient elution method was used to separate and detect the concentration of the sample to be measured. The elution conditions were set as showed in Table S2. In addition, mobile phase A is acetonitrile, mobile phase B is a mixture of acetic acid glacial and water, and the flow rate is 1 mL/min. Filter through 0.22 μm filter before injection.

S3.4. Drawing of standard curves

The peak area (horizontal coordinate) obtained by HPLC and the measured

standard solution concentration (g/mL) (vertical coordinate) were linearly fitted, and the fitting standard curves of ECH and ACT were obtained. Table S3 lists the results.

S3.5. Precision test

The same sample was injected 5 times for accuracy testing, and experimental results showed that the peak area standard deviation (RSD) of ECH and ACT were 1.23% and 1.61%, respectively, indicating good precision of the detector.

S3.6. HPLC diagram of ECH and ACT

HPLC diagrams of ECH and ACT are showed in Figure S2. It can be concluded that both ECH and ACT can be detected at 330 nm wavelength, and their peak locations are 13 min and 21 min, respectively.

S4. Performance test of adsorbents

S4.1. Testing of adsorption properties

10 mg of the adsorbent was added to 15 mL of the prepared crude extract sample solution, and the adsorption experiment was carried out in a constant temperature shaking table. The adsorption conditions were as follows: the temperature was 303 K and the rotational speed was 150 r/min. After 24 h, the conical bottle was removed, passed through 0.22 μm filter, and the concentration of ECH and ACT in the filtrate was determined by HPLC. Adsorption capacity (Q, mg/g) and selectivity (S) were used as the criteria to evaluate the adsorption performance of adsorbents. The formula was as follows:

$$Q = \frac{(C_0 - C_e)V}{m} \tag{S1}$$

$$S = \frac{Q_{ACT}}{Q_{ECH}} \quad (S2)$$

Where, C_0 (mg/mL) represents the initial concentration of ACT and ECH, and C_e (mg/mL) represents the equilibrium concentration of ACT and ECH. Q_{ACT} (mg/g) and Q_{ECH} (mg/g) represent the adsorption capacity of ACT and ECH in equilibrium, respectively. V (mL) represents the volume of the adsorption solution; m (mg) indicates the mass of the adsorbent.

S4.2. Adsorption kinetics

100mg of adsorbent was added to 150 mL of prepared crude extract sample solution. The conical bottle containing the adsorption solution was placed in a constant temperature shaking table (temperature 303 K, speed 150 r/min) for adsorption experiment for 2 h (parallel 3 groups). In this process, HPLC was used to detect the concentration of ACT at different times of 5-120 min, and the calculation formula was showed in (S3). In addition, the adsorption kinetics data were simulated by a pseudo-first-order kinetic model (S4), a pseudo-second-order kinetic model (S5), a Ritchie-second-order model (S6), and an intra-particle diffusion model (S7).

$$Q_t = \frac{(C_0 - C_t)V}{m} \quad (S3)$$

$$Q_t = Q_e\left(1 - e^{-k_1 t}\right) \quad (S4)$$

$$Q_t = \frac{k_2 Q_e^2 t}{(1 + k_2 Q_e t)} \quad (S5)$$

$$\frac{1}{Q_t} = \frac{1}{k_3 Q_e t} + \frac{1}{Q_e} \quad (S6)$$

$$Q_t = K_3 t^{1/2} + C \quad (S7)$$

Among them, Q_t (mg/g) and Q_e (mg/g) represent at t (min) the adsorption capacity and the adsorption capacity at equilibrium, respectively. C_0 (mg/mL) and C_t (mg/mL) represent the concentration at initial concentration and t time, respectively. k_1 (min^{-1}), k_2 (g/mg/mL) and k_3 (min^{-1}) represent the rate constants of the pseudo-first-order, pseudo-second-order and Ritchie-second-order kinetic models, respectively. K_3 (mg/min$^{1/2}$·g) represents the rate constant of intra particle diffusion. C is a constant.

S4.3. Adsorption isotherm and thermodynamics

The 10 mg adsorbent was added into the crude extract sample solution prepared with different concentrations. Adsorption experiments were performed by placing a conical bottle containing the sample solution in a constant temperature shaker (temperature 303 K, rotational speed 150 r/min) (parallel 3 sets). After the shaker was stopped, the conical bottle was removed and the concentration of ACT and ECH was determined by HPLC through 0.22 μm filter. At the same time, the obtained data were fitted by Freundlich isotherm model (S8), Langmuir isotherm model (S9), and Liu isotherm model (S10). Enthalpy change (ΔH), entropy change (ΔS) and Gibbs free energy change (ΔG) (S11-13). Calculate by the following formula.

$$Q_e = K_F C_e^{1/n} \qquad \text{(S8)}$$

$$Q_e = \frac{Q_m K_L C_e}{1 + K_L C_e} \qquad \text{(S9)}$$

$$\frac{1}{Q_e} = \frac{1}{Q_m (K_{Liu} C_e)^{n_{Liu}}} + \frac{1}{Q_m} \qquad \text{(S10)}$$

$$K_d = \frac{q_e}{C_e} \qquad \text{(S11)}$$

$$\ln K_d = \frac{\Delta S}{R} + \frac{-\Delta H}{RT} \quad \text{(S12)}$$

$$\Delta G = \Delta H - T\Delta S \quad \text{(S13)}$$

Where, Q_e (mg/g) represents the equilibrium adsorption capacity of the adsorbent for ACT. C_e (mg/mL) represents the equilibrium concentration; K_F stands for Friedrich's constant; n stands for dimensionless Flanders constant; Q_m (mg/g) represents the maximum adsorption capacity; K_L (mL/mg) represents Langmuir's constant; K_{Liu} (mL/mg) stands for Liu equilibrium constant; n_{Liu} represents the dimensionless exponent of the Liu equation. K_d represents the partition coefficient; R stands for gas constant (8.314 J/mol·K); T (K) is the absolute temperature. ΔH and ΔG have the relationship between 1/T and $\ln K_d$ by calculating the slope of the curve. ΔH and ΔG are calculated from the slope of the curve of the relationship between 1/T and $\ln K_d$.

S4.4. Cycle performance test

The adsorbent after adsorption was filtered out for elution regeneration experiment. The desorption solution is prepared by methanol, acetic acid and ultra-pure, and then the filtered adsorbent is added to the conical bottle, the desorption solution is added for desorption and regeneration, and the sample is obtained, washed with ultra-pure water and dried in vacuum. Continue the new adsorption experiment. Repeat the preceding steps eight times.

S5. Supplementary Figures

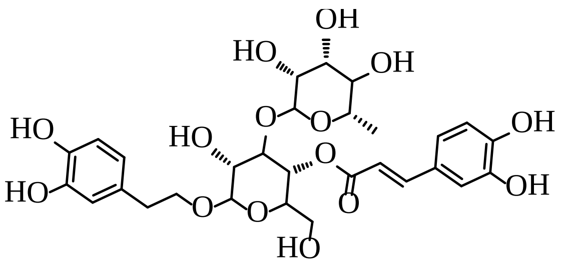

Figure S1. The structural formula of ACT.

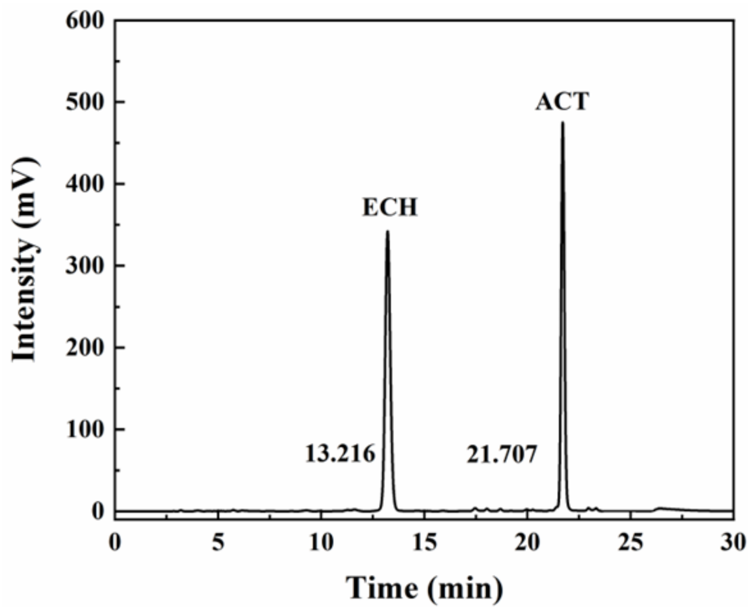

Figure S2 HPLC chromatogram of ACT and ECH.

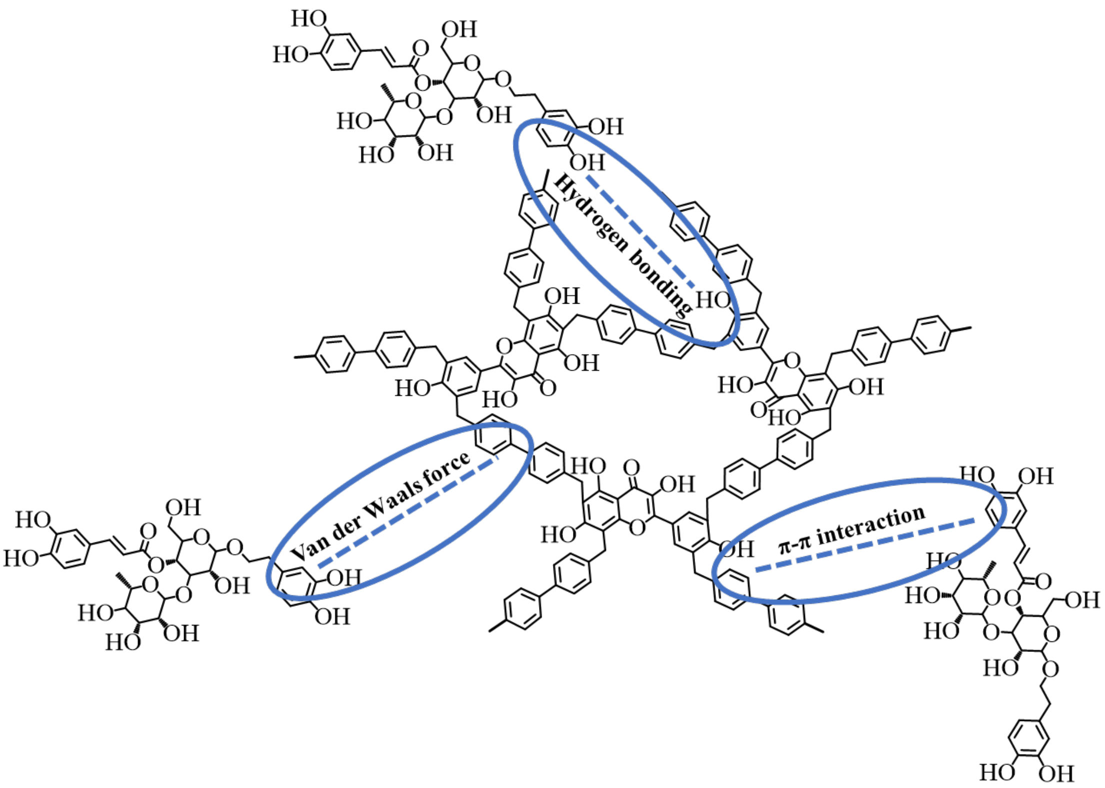

Figure S3 The main interaction between BHP-Kae-3 and ACT.

.

S5. Supplementary Tables

Table S1 Specific surface area parameters and pore structure of BHP-Kae.

Adsorbents	Pore size (nm)	Pore volume (cm^3/g)	Specific surface area (m^2/g)
BHP-Kae-1	15.78	0.007	18.62
BHP-Kae-2	16.69	0.010	21.12
BHP-Kae-3	17.07	0.141	82.36
BHP-Kae-4	16.78	0.081	32.82

Table S2 The gradient elution condition of HPLC

Time (min)	Mobile phase A (%)	Mobile phase B (%)
0~8	13	87
8~20	13~20	87~80
20~23	20~50	80~50
23~25	50~13	50~87
25~30	13	87

Table S3 The calibration curves of ACT and ECH

Ingredient	Linear equation	R^2
ECH	$Y=1.81\times10^{7}X-2.8\times10^{4}$	0.99999
ACT	$Y=1.79\times10^{7}X+1.5\times10^{5}$	0.99976

Table S4 Kinetics parameters of ACT adsorption on BHP-Kae-3.

Adsorbent	Pseudo-first-order model			Pseudo-second-order model			Ritchie-second-order model		
	Q_e (mg/g)	K_1	R^2	Q_e (mg/g)	K_2	R^2	Q_e (mg/g)	K_3	R^2
BHP-Kae-3	68.05	0.17	0.9693	112.87	0.0019	0.9991	119.90	0.1487	0.9878

Table S5 Intra-molecule diffusion coefficient of ACT on BHP-Kae-3.

	k_1 (mg/g·h$^{1/2}$)	C_1 (mg/g)	R^2	k_2 (mg/g·h$^{1/2}$)	C_2 (mg/g)	R^2	k_3 (mg/g·h$^{1/2}$)	C_3 (mg/g)	R^2
ACT	45.35	24.13	0.98	15.99	46.74	0.99	2.64	89.92	0.98

Table S6 Adsorption isotherm parameters for BHP-Kae-3.

Temperature	Freundlich model			Langmuir model			Liu model		
(K)	K_F	1/n	R^2	Q_m (mg/g)	K_L	R^2	K_{liu}	n	R^2
293	136.45	0.4720	0.9307	229.87	1.57	0.9821	2.77	1.48	0.9961
303	115.44	0.4408	0.9355	184.31	1.81	0.9817	2.19	1.21	0.9975
313	100.63	0.4232	0.9104	155.86	1.98	0.9668	1.91	1.20	0.9973

Table S7 Thermodynamic parameters of ACT adsorption.

Temperature (K)	$\varDelta G$ (kJ/mol)	$\varDelta H$ (kJ/mol)	$\varDelta S$ (J /mol•K)
293	-5.778	-1.843	13.425
298	-5.846	–	–
303	-5.913	–	–
308	-5.983	–	–

313	-6.047	–	–

Table S8 Comparing the adsorption capacities of various ACT adsorbents

Adsorbents	Pore volume (m^3/g)	Pore size (nm)	Adsorption capacity (mg/g)	Selectivity
Fe_3O_4 @MCM-41-MIPs[S1]	0.298	17.0	1.63	-
NADES[S2]	-	-	2.13	2.89
HPD300[S3]	-	5-5.5	40.35	1.12
[Bemim]Cl[S4]	-	-	0.92	-
HSCCC[S5]	-	-	10.34	-
BHP-Kae-3	0.141	17.07	105.12	3.41

References

[S1]Li C, Nie F, Feng C, Tian M, Yu M, Zhao C, Fu Y. Magnetic dual-template molecularly imprinted polymers for separation and enrichment of echinacoside and acteoside from Cistanche deserticola Y. C. Ma[J]. Chemical Engineering Research and Design, 2022, 182: 719–732.

[S2]Nie F, Feng C, Ahmad N, Tian M, Liu Q, Wang W, Lin Z, Li C, Zhao C. A new green alternative solvent for extracting echinacoside and acteoside from Cistanche deserticola based on ternary natural deep eutectic solvent[J]. Journal of Industrial and Engineering Chemistry, 2023, 118: 499–510.

[S3]Liu B, Ouyang J, Yuan X, Wang L, Zhao B. Adsorption properties and preparative separation of phenylethanoid glycosides from Cistanche deserticola by use of macroporous resins[J]. Journal of Chromatography B, 2013, 937: 84–90.

[S4]Fan Y, Xu C, Li J, Zhang L, Yang L, Zhou Z, Zhu Y, Zhao D. Ionic liquid-based microwave-assisted extraction of verbascoside from Rehmannia root[J]. Industrial Crops and Products, 2018, 124: 59–65.

[S5]Li L, Tsao R, Yang R, Liu C, Young J C, Zhu H. Isolation and purification of phenylethanoid glycosides from Cistanche deserticola by high-speed counter-current chromatography[J]. Food Chemistry, 2008, 108(2): 702–710.

19
